# Towards next-generation diagnostic pathology: AI-empowered label-free multiphoton microscopy

**DOI:** 10.1038/s41377-024-01597-w

**Published:** 2024-09-14

**Authors:** Shu Wang, Junlin Pan, Xiao Zhang, Yueying Li, Wenxi Liu, Ruolan Lin, Xingfu Wang, Deyong Kang, Zhijun Li, Feng Huang, Liangyi Chen, Jianxin Chen

**Affiliations:** 1 School of Mechanical Engineering and Automation, Fuzhou University, Fuzhou, 350108 China; 2 Key Laboratory of OptoElectronic Science and Technology for Medicine of Ministry of Education, Fujian Provincial Key Laboratory of Photonics Technology, Fujian Normal University, Fuzhou, 350007 China; 3 College of Computer and Data Science, Fuzhou University, Fuzhou, 350108 China; 4 Department of Radiology, Fujian Medical University Union Hospital, Fuzhou, 350001 China; 5 Department of Pathology, The First Affiliated Hospital of Fujian Medical University, Fuzhou, 350005 China; 6 Department of Pathology, Fujian Medical University Union Hospital, Fuzhou, 350001 China; 7 New Cornerstone Laboratory, State Key Laboratory of Membrane Biology, Beijing Key Laboratory of Cardiometabolic Molecular Medicine, Institute of Molecular Medicine, National Biomedical Imaging Center, School of Future Technology, Peking University, Beijing, 100091 China

**Keywords:** Biophotonics, Multiphoton microscopy

## Abstract

Diagnostic pathology, historically dependent on visual scrutiny by experts, is essential for disease detection. Advances in digital pathology and developments in computer vision technology have led to the application of artificial intelligence (AI) in this field. Despite these advancements, the variability in pathologists’ subjective interpretations of diagnostic criteria can lead to inconsistent outcomes. To meet the need for precision in cancer therapies, there is an increasing demand for accurate pathological diagnoses. Consequently, traditional diagnostic pathology is evolving towards “next-generation diagnostic pathology”, prioritizing on the development of a multi-dimensional, intelligent diagnostic approach. Using nonlinear optical effects arising from the interaction of light with biological tissues, multiphoton microscopy (MPM) enables high-resolution label-free imaging of multiple intrinsic components across various human pathological tissues. AI-empowered MPM further improves the accuracy and efficiency of diagnosis, holding promise for providing auxiliary pathology diagnostic methods based on multiphoton diagnostic criteria. In this review, we systematically outline the applications of MPM in pathological diagnosis across various human diseases, and summarize common multiphoton diagnostic features. Moreover, we examine the significant role of AI in enhancing multiphoton pathological diagnosis, including aspects such as image preprocessing, refined differential diagnosis, and the prognostication of outcomes. We also discuss the challenges and perspectives faced by the integration of MPM and AI, encompassing equipment, datasets, analytical models, and integration into the existing clinical pathways. Finally, the review explores the synergy between AI and label-free MPM to forge novel diagnostic frameworks, aiming to accelerate the adoption and implementation of intelligent multiphoton pathology systems in clinical settings.

## Abstract (web summary)

AI-empowered multiphoton microscopy enhances diagnostic accuracy and efficiency for various human diseases, evolving towards next-generation diagnostic pathology with an endogenous, multi-dimensional, and intelligent approach.

## Graphical abstract

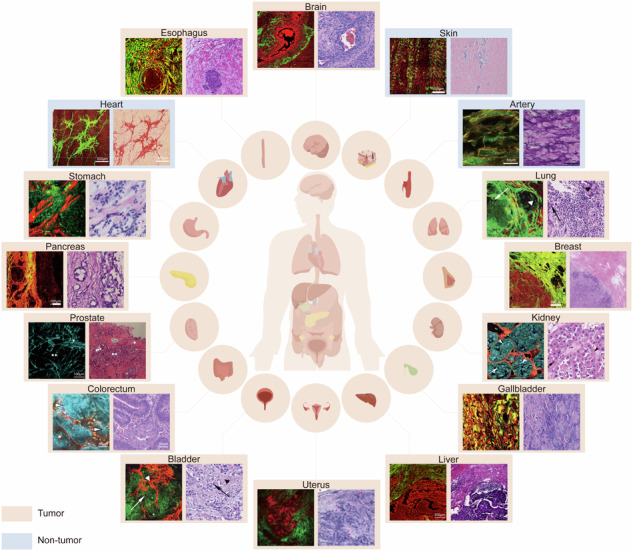

## Introduction

Pathology often provides the “gold standard” for disease diagnosis^1^. Historically, this discipline has relied on the keen eyes of pathologists to make clinical judgments based on visual examinations of stained tissue sections under a microscope to classify diseases and determine their prognoses. The advent of whole slide imaging (WSI) scanners in the last decade has transformed how these images are collected and examined, ushering in a new era for pathology^2^. WSIs have become a cornerstone for remote pathology consultations, routinely facilitating diagnosis, research, and education in pathology, offering unprecedented convenience to practitioners. However, the reliance on the extensive expertise of experienced pathologists persists, whether reviewing slides under a microscope or analyzing WSIs. The training cycle for professional pathologists remains lengthy and demanding. This factor, coupled with the growing number of cases needing diagnosis annually, poses an escalating challenge for medical institutions striving to maintain high-quality diagnostic services.

Artificial intelligence (AI), predominantly driven by deep learning, has shown its superiority in various computer vision applications including image enhancement^3–5^, classification^6^, detection^7^, and segmentation by automatically recognizing and extracting complex features from images^8^. Simultaneously, the availability of large-scale WSI datasets rich in pixel-level detail has allowed the expansion of deep learning techniques, traditionally applied to natural images, to the realm of microscopic imagery. The digital transformation of clinical pathology has led to the automation of various aspects of the field, integrating supportive diagnostic techniques such as diagnosis^9^, biomarker identification^10^, and prediction^11^, which combinedly to be computational pathology.

The WSI-based intelligent pathology not only alleviates the burden on pathologists but also provides both patients and clinicians with more objective tools for diagnosis and prognosis. Nevertheless, diagnostic procedures within pathology still face a “gray zone”, where varying interpretations of diagnostic criteria among pathologists can lead to discrepancies in the diagnosis of certain conditions. The precision required for personalized cancer therapy further escalates the need for accurate tissue pathology marker diagnosis, whereas misdiagnoses can lead to misguided treatments and can also hinder the progress of drug development. Consequently, to develop a new generation of pathological diagnostic paradigms, it is essential not only to create an AI-assisted diagnostic framework but also to incorporate innovative multimodal imaging techniques that enhance conventional pathology.

The most commonly pathological staining method is hematoxylin and eosin (H&E) staining. Irrespective of intraoperative frozen sections or postoperative paraffin sections, the production of H&E slides involves intricate histological procedures such as biopsy, fixation, sectioning, and staining. With the advancement of label-free optical microscopy^12,13^, techniques such as quantitative phase imaging (QPI)^14,15^, photoacoustic microscopy (PAM), optical coherence tomography (OCT)^16^, and stimulated Raman scattering (SRS) microscopy have complemented traditional pathology. They offer unique insights into cellular physical parameters in vitro, functional imaging in vivo, and tissue molecular characteristics. Notably, multiphoton microscopy (MPM) enables simultaneous imaging of multiple intrinsic components within biological tissues. Moreover, it attains imaging contrast and resolution comparable to traditional histopathology, directly extracting qualitative microstructure and quantitative spectral features for pathological diagnosis^17^. Enabled by deep learning methodologies, OCT facilitated the automated detection of geographic atrophy in age-related macular degeneration^18^. QPI allowed for virtual quantitative fluorescent imaging of live organoids^19^, while PAM allowed intraoperative histology of bone tissue^20^. Additionally, SRS provided near real-time intraoperative diagnosis of brain tumors, creating a complementary diagnostic pathway independent of traditional pathology laboratories^21^. These technologies significantly enhance the accuracy and efficiency of diagnosis. For AI-empowered MPM, comprehensive exploration of multiphoton feature patterns, such as tumor infiltration patterns^22–25^ and vascular collagen deposition^17,26,27^, has been integrated to achieve distinctive auxiliary diagnosis^28–41^ and prognosis prediction^42^ capabilities, holding great promise for clinical translation.

In this review, we first provide a concise overview of multiphoton physics mechanisms and multiphoton microscopic instrument. Subsequently, we systematically summarize pathological applications of MPM in various human diseases. Drawing on multiphoton pathological imaging, we explore the positive impact of artificial intelligence — extending from machine learning to deep learning — in advancing diagnostics assisted by multiphoton pathology. Finally, considering the current status of multiphoton intelligent pathology and the requirements for precision diagnostics, we discuss the challenges and future perspectives associated with the integration of MPM and AI. We anticipate that this review will contribute to the clinical translation and intelligent applications of multiphoton microscopy, fostering progress in “next-generation diagnostic pathology”.

## Label-free multiphoton microscopy

Label-free optical microscopy exploits the interaction of light with biological tissues, such as refractive index, molecular vibrations, scattering, or absorption, to achieve various imaging contrasts. Table 1 provides a comparative overview of the capabilities and applications of common label-free biomedical microscopy. The diverse imaging mechanisms of these techniques render them suitable for different clinical applications. QPI measures phase changes to obtain contour and morphology information of in vitro cell samples. This computational-based optical system is both simple and cost-effective. PAM and OCT achieve imaging depths at the millimeter scale. Although they sacrifice some spatial resolution, there in vivo vasculature and ophthalmology applications are also clinically recognized.

**Table 1:** Comparison of label-free biomedical microscopy

Method	Imaging mechanism	Main endogenous signal source	Spatial resolution	Imaging depth	Label-free capabilities complementary to H&E staining	Potential main clinical applications
Multiphoton microscopy (MPM)	Second/third harmonic generation (SHG/THG); Two/three photon excited autofluorescence (TPEF/3PEF)	SHG: non-centrosymmetric structures (e.g., collagen fibers, elastin fibers, microtubules); THG: interfaces and optical heterogeneities (e.g., lipid-based biological membranes); TPEF/3PEF: cellular and metabolic markers (e.g., NAD(P)H, FAD, porphyrin)	Submicrometre	Submillimetre	Specific imaging of collagen and elastic fiber; Multichannel dynamic observation of tumor microenvironment	Ex vivo pathological diagnosis; In vivo cellular metabolism detection
Stimulated Raman scattering microscopy (SRS)	Raman scattering of molecular vibrations	Molecule or chromophore (e.g., lipids, proteins, DNA)	Submicrometre	Submillimetre	High chemical selectivity providing molecular chemical information	Ex vivo pathological diagnosis; Chemical component analysis
Quantitative phase imaging (QPI)	Interference between incident and scattered waves	Refractive index in tissue structures	Submicrometre	Tens of micrometers	Quantitative measurement of physical parameters of cells and tissues	In vitro cell imaging and dynamics
Photoacoustic microscopy (PAM)	Photoacoustic effect	Endogenous chromophores (e.g., hemoglobin, myoglobin, melanin, water, lipids, nucleic acids)	submicrometre to tens of micrometers	submillimetre to tens of millimeters	Deep tissue functional imaging; Providing functional information such as oxygen saturation	In vivo cerebrovascular and cardiovascular imaging
Optical coherence tomography (OCT)	Low-coherence interferometry	Variations in the backscattering cross-section of various tissue components	Several micrometers	Several millimeters	Non-contact real-time three-dimensional tomographic imaging	In vivo ophthalmic imaging; Dermatopathology

To obtain high-resolution, high-contrast images resembling those produced by traditional pathology, two nonlinear optical microscopies, MPM and SRS have been widely applied in label-free pathological diagnosis. SRS not only acquires pathological images but also enables selective Raman spectral analysis of components such as lipids and proteins. However, the complexity of the excitation light source module in current SRS systems has hindered its widespread commercial adoption, and further exploration is needed to fully establish its indications for pathological diagnosis. In contrast, commercial multiphoton microscopes, based on SHG and TPEF, have reached a high level of maturity and availability since their inception in 1997. MPM has been applied to examine tumor pathology in as many as 16 human organs, such as brain tumors^17,43–47^, breast cancer^23,24,48–53^, and colorectal cancer^30,54–57^. Consequently, MPM was highlighted as one of the significant advancements in label-free histopathology in the 2016 research highlights of Nature Methods^58^.

### The principle of multiphoton microscopy

MPM requires high peak power from ultra-short pulse lasers. To capture a multiphoton image of a single field of view, the excitation light scans the specimen point-by-point and line-by-line via scanning system and objective. When multiple low-energy photons simultaneously reach the fluorophores or specific structures in specimen, they interact to produce multiphoton optical signals, including two-photon/three-photon excited fluorescence and second/third harmonic generation. These signals are typically collected in an epi-detection configuration by the objective and guided onto the photomultiplier tubes, which convert the optical information into electrical signals. By utilizing an XY translation stage to sequentially capture images from each position within the specimen, a large-scale stitched image can be constructed.

### TPEF

TPEF is a third-order nonlinear absorption process. In this process, a fluorescent molecule or atom simultaneously absorbs two photons of the same frequency. During the absorption process, electrons in the ground state are first excited to an intermediate “virtual state” by one photon and then further excited to the final excited state by another photon. In other words, absorption of two photons of the same frequency excites electrons to a higher energy level. Following a certain relaxation time, electrons in the excited state spontaneously transition back to the ground state, emitting fluorescence with a frequency slightly lower than twice the incident light frequency.

### SHG

SHG is a second-order nonlinear optical phenomenon, also known as “frequency doubling”. It refers to the output photons having twice the frequency of the incident photons when two photons of the same frequency interact with a nonlinear medium. The output second harmonic wave is termed the second harmonic. In the process of second harmonic generation, an electron in the ground state absorbs two photons of the same frequency, is excited to a virtual state, and then emits a second harmonic photon before returning to the ground state.

### Endogenous signal sources

**Fig. 1:**
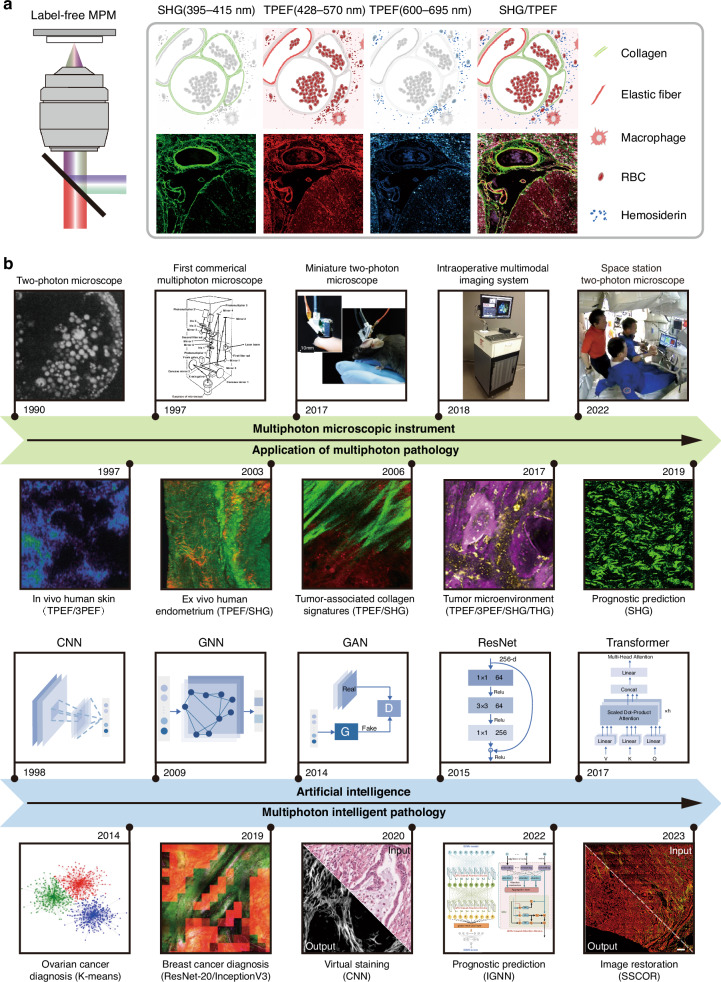
The development history of AI-empowered label-free multiphoton microscopy. **a** Schematic diagram of label-free multiphoton imaging, representative SHG/TPEF images, and corresponding endogenous signal sources^17^. **b** Historical timeline of multiphoton microscopic instruments^52,65–68^, multiphoton pathological applications^25,27,69–71^, and AI-empowered multiphoton intelligent pathology^31,41,42,72,73^. TPEF two-photon excited fluorescence, 3PEF three-photon excited fluorescence, SHG second-harmonic generation, THG third-harmonic generation

In biological tissues, numerous biomolecules exhibit TPEF and SHG signals. For instance, TPEF can image endogenous fluorophores such as nicotinamide adenine dinucleotide (NADH) and flavin adenine dinucleotide (FAD)^59^. SHG occurs in non-centrosymmetric molecular structures like collagen^60^, microtubules^61^, and myosin^62^. Thus, SHG and TPEF endogenous signals provides a comprehensive characterization of tissue structure and multi-parameter functional metabolism. This approach overcomes the influence of labeled biological processes or toxicity, offering a crucial tool for studying pathological samples. Taking the example of multiphoton imaging of cerebral vascular malformations, Fig. 1a illustrates images from the SHG and two TPEF detection channels, along with a schematic representation of endogenous signal sources^17^. Detailed endogenous signal sources have been summarized in previous references^63,64^.

## Multiphoton microscopic instrument

Figure 1b illustrates the representative history of AI-empowered label-free MPM^65–73^. In 1931, Maria Goeppert-Mayer proposed the concept of TPEF^74^. Thirty years later, the invention of the laser facilitated the first experimental verification of TPEF^75^. In 1974, Robert Hellwarth introduced the SHG microscope, utilized for observing spatial structural changes in ZnSe crystals^76^. In 1990, the Webb group introduced the concept of two-photon excitation fluorescence microscopy, marking DNA in pig kidney cells and observing chromosome morphology in live cells^65^. In 1997, the Bio-Rad company produced the first commercial multiphoton laser scanning microscope. Currently, globally microscope companies are continually innovating desktop multiphoton laser scanning microscopy, greatly advancing the life sciences^77,78^.

The commercial research-grade multiphoton microscopes, designed to meet the needs of most researchers, typically can simultaneously image both labeled and unlabeled specimens. However, the large equipment footprint of such microscopes requires placement on laboratory optical platforms, and their high cost discourages some users. Therefore, researchers have been devoted to developing more portable and economical multiphoton microscopes. Although the miniaturized design and integration technology may sacrifice image resolution or field of view, this trade-off makes the instrument more portable, suitable for applications in on-site pathology diagnosis and other widespread diagnosis scenarios. In 2017, fast high-resolution miniature two-photon microscopy was successfully applied to brain imaging in freely behaving mice^67^. In 2018, the multimodal label-free nonlinear imaging system was implemented to intraoperatively characterize the tumor microenvironment^52^. Excitingly, in 2023, the space station-level two-photon microscope achieved the first three-dimensional images of astronauts’ skin. For future challenges in the development of multiphoton microscopic instrument from research-grade to pathological-grade, please refer to Section 6.3.

With the continuous development of multiphoton instruments, there has been a significant emergence of pathological applications in MPM. Meanwhile, the rise of artificial intelligence technology enables multiphoton intelligent pathology. Section 4 introduces the applications of multiphoton pathology, while Section 5 focuses on AI-empowered multiphoton pathology diagnosis.

## Applications of multiphoton microscopy in pathological diagnosis

Label-free MPM, with its specific identification of cellular cytoplasm, extracellular matrix, and their interactions, has opened a novel perspective in pathological research. This section summarizes the applications of MPM in pathological diagnosis through the exploration of multiphoton diagnostic features.

Firstly, multiphoton imaging of the cytoplasm reveals rich cellular morphological information, such as cancer cells^79^, hyperplasia^43,55^, and necrosis^44^, which is crucial for determining the grading and prognosis of tumors. Additionally, through the analysis of specific features of cancer nests, different tumor growth patterns^80^ can be distinguished, providing a basis for the formulation of clinical treatment plans. It is noteworthy that MPM can also quantitatively reflect cellular metabolic activity by measuring the ratio of NADH to FAD in the cytoplasm^81^. In addition to cancer cell identification, MPM can differentiate other cell types, such as myoepithelial cells^82^, lymphocytes^37^, and glandular cells^83^, based on differences in cytoplasmic morphology and signal intensity. Taking myoepithelial cells as an example, this provides crucial features for challenging diagnoses such as microinvasive breast cancer.

Secondly, MPM exhibits high sensitivity to the extracellular matrix, especially collagen fibers^84^ and basement membrane^80^. By analyzing the morphology of collagen fibers, different vascular patterns in tumors can be distinguished^43^, aiding in the assessment of malignancy and progression of tumors. For instance, observations of glomeruloid vessels in glioblastomas^85^, hyaline degeneration and collagen aging in cerebral cavernous malformations^17^. Furthermore, the quantification of fibrosis^45^ and proliferative reactions^86^ can be facilitated by extracting features of collagen fibers, which provides crucial evidence for disease progression. More importantly, combining information from the cytoplasm and extracellular matrix, MPM can observe diverse spatial distribution patterns, such as tumor-associated collagen signatures (TACS)^23^ and tumor-infiltrating lymphocytes (TILs)^24^, offering a unique perspective on the occurrence and development of infiltrating tumors such as gastric cancer, colorectal cancer, and breast cancer.

**Fig. 2:**
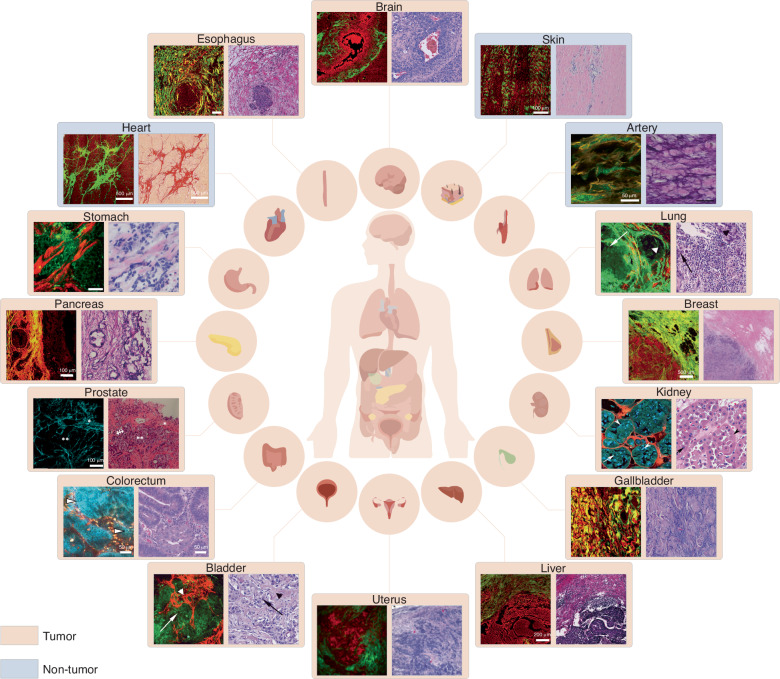
Representative multiphoton pathological atlas of different diseases. Multiphoton microscopy can be used for a variety of diseases in different tissues of the human body. These include tumor diseases such as glioblastoma, liver cancer^37^, hilar cholangiocarcinoma^95^, breast cancer^23^, ovarian cancer peritoneal metastases^94^, prostate cancer^93^, colorectal cancer^54^, oncocytoma^90^, intramural metastasis in esophageal squamous cell carcinoma^91^, ductal adenocarcinoma in the pancreatic head^92^, gastric cancer^89^, nonpapillary urothelial carcinoma^88^, and squamous cell carcinoma of the lung^87^. Additionally, it can be utilized for non-tumor conditions like morphea^28^, myocardial fibrosis^97^, and atherosclerotic lesions^96^. Schematic diagram of human body structure and organs are drawn by Figdraw. Adapted with permission from ref. ^91,94^ © Optical Society of America

**Fig. 3:**
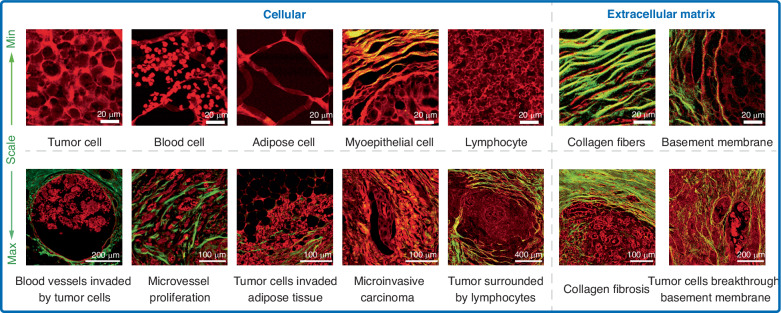
Typical multiphoton pathological diagnostic features of breast cancer. Multiphoton microscopy can identify cellular-level features, such as tumor cells, blood cells, adipose cells, myoepithelial cells, and lymphocytes on a small scale. These correspond to phenomena such as blood vessels invaded by tumor, microvessel proliferation^98^, tumor invasion of adipose tissue, microinvasive carcinoma^82^, and tumors surrounded by lymphocytes^24^ on a larger scale. Additionally, it is possible to identify extracellular matrix-level features, such as collagen fibers and basement membrane on a small scale, which correspond to collagen fibrosis^99^ and tumor breakthrough the basement membrane^23^ on a larger scale

Figure 2 presents a representative multiphoton pathological atlas of different diseases (2013–2023), encompassing both tumor^23,37,54,87–95^ and non-tumor components^28,96,97^. We prioritized articles that included corresponding pathological staining images for multiphoton images. Figure 3 illustrates typical multiphoton diagnostic features of breast cancer^98,99^. Besides, Table 2 provides a detailed summary of the imaging parameters and typical multiphoton pathological characteristics of MPM applied to both tumor^100–114^ and non-tumor^115–131^ diseases.

**Table 2:** (a) Multiphoton imaging parameters and pathological characteristics of tumor diseases. (b) Multiphoton imaging parameters and pathological characteristics of non-tumor diseases

Organs	Authors	Disease	Specimen	Excitation wavelength (nm)	Pulse width (fs)	Repetition frequency (MHz)	Detection wavelength		Representative multiphoton pathological characteristics
							TPEF (nm)	SHG (nm)	
**(a)**									
Brain	Wang et al. ^17^	Cerebral cavernous malformation	Frozen section	810	140	80	428–570, 600–695	395–415	1. **Cell morphology:** cellular pleomorphism, increased nuclear size, non-polarized nuclei, disordered cellular arrangement, higher nuclear-cytoplasmic ratio, irregular cell size and shape, cells with mucinous cytoplasm, homogeneous cell population with rich cytoplasm, multilayered cells, and umbrella-shaped surface cells.
	Fang et al. ^43^	Glioma	Frozen section	810	110	76	430–716	387–419	
	Mehidine et al. ^44^	Glioblastoma metastasis	Resected tissue	890	70	80	505–535	438–458	
	Fang et al. ^45^	Intracranial germinoma	Frozen section	810	140	80	430–690	395–415	
	Fang et al. ^46^	Schwannoma	Frozen section	810	110	76	430–716	389–419	
	Lin et al. ^47^	Pituitary adenoma	Frozen section, paraffin section	810	110	76	430–716	389–419	
Laryngeal	Zhang et al. ^83^	Laryngeal squamous cell carcinoma	Resected tissue	900–1200	40	13.6	3PEF: 415–526	SHG: 530–570; THG: 340–380	2. **Tissue structure:** tumor-infiltration lymphocytes, tumor necrosis, glandular-tubular structures, hemosiderin-related products, changes or fusion in glandular morphology, intercellular bridges, formation of keratin pearls, densely packed elongated cells with bundle or vortex arrangement, disrupted ductal structures, and a mixture of tumor cell nests. 3. **Stroma:** blood vessels: vascular proliferation, vascular malformation, clustering of lymphocytes around blood vessels, collagen deposition around blood vessels, and proliferation or cysts of vascular Brunn nests. Collagen: collagen fibers in a disorganized and fragmented state, entwined around the tumor ducts, collagen fibers surrounding glandular tissues, collagen deposition around blood vessels, early alterations in fibrous collagen and elastic fiber networks, continuous damage to muscular tissues, aggregation of collagen fibers into bundles, and tumor-associated collagen signatures.
Esophagus	Zeng et al. ^84^	Esophageal submucosal cancer	Frozen section	810	110	76	430–716	387–419	
	Xu et al. ^91^	Esophageal squamous cell carcinoma	Frozen section	810	110	76	430–710	387–419	
Lung	Xi et al. ^100^	Early-stage lung adenocarcinoma	Frozen section	810	140	80	428–695	395–415	
	Golaraei et al. ^101^	Non-small cell lung carcinoma	Frozen section	1028	430	14.3	N/A	510–520	
	Jain et al. ^87^	Invasive and preinvasive adenocarcinoma, squamous cell carcinoma	Resected tissue	780	N/A	N/A	420–490, 550–650	360–400	
Breast	He et al. ^48^	Invasive micropapillary breast carcinoma	Paraffin section	810	140	80	428–695	395–415	
	Han et al. ^49^	Invasion breast tumor underwent preoperative chemotherapy	Paraffin section	810	140	80	430–759	394–416	
	Xi et al. ^23^	Invasive breast cancer	Paraffin section	810	140	80	428–695	395–415	
	He et al. ^24^	Oestrogen receptore-positive breast cancer	Paraffin section	810	140	80	428–695	395–415	
	Gavgiotaki et al. ^50^	Ductal carcinoma	Paraffin section	1028	200	50	690–710, 400–517, 548–710	SHG: 509–519; THG: 297.5–382.5	
	Shen et al. ^51^	Lobular breast carcinoma	Frozen section	810	140	80	428–695	395–415	
	Sun et al. ^52^	Invasive ductal carcinoma, ductal carcinoma in situ	Resected tissue	1070	55	70	TPEF: 590–740; 3PEF: 420–470	SHG: 510–560; THG: 330–380	
	Nie et al. ^53^	Fibroadenoma	Frozen section	810	110	76	430–716	389–419	
Stomach	Li et al. ^102^	Early gastrointestinal, neuroendocrine tumors	Frozen section	810	110	76	430–716	389–419	
	Zheng et al. ^103^	Mucosal adenocarcinoma	Resected tissue	810	110	76	430–716	387–419	
	Chen et al. ^27^	Early gastric cancer	Paraffin section	810	110	76	430–708	387–409	
	Yan et al. ^89^	Gastric cancer with serosal invasion	Resected tissue	800	110	76	430–708	387–410	
Colorectum	Terradillos et al. ^30^	Malignant neoplastic colon lesion	Paraffin section	785	N/A	80	N/A	N/A	
	Matsui et al. ^54^	Colorectal carcinoma	Resected tissue	780	N/A	N/A	387–447, 460–500, 601–657	N/A	
	Li et al. ^55^	Neoadjuvant Therapy for rectal adenoma carcinoma	Frozen section	810	110	76	430–716	387–419	
	Li et al. ^56^	Rectal carcinoma following preoperative radiochemotherapy	Frozen section	810	110	76	430–716	387–419	
	Yan et al. ^57^	Low rectal cancer	Resected tissue	800	110	76	430–716	387–419	
Liver	Huang et al. ^37^	Liver fibrosis, early hepatocellular carcinoma	Paraffin section	810	110	76	430–759	394–416	
	Lin et al. ^104^	Hepatocellular carcinoma	Paraffin section	810	110	76	430–690	400–410	
	Yan et al. ^79^	Benign and malignant liver lesions	Resected tissue	800	110	76	430–708, 430–490, 500–560	387–409	
Gallbladder	Zhan et al. ^95^	Hilar cholangiocarcinoma	Frozen section	810	140	80	430–650	390–420	
Kidney	Jain et al. ^90^	Oncocytoma, chromophobe renal cell carcinoma	Paraffin section	780	N/A	N/A	420–490, 550–650	360–400	
	Chen et al. ^105^	Pancreatic metastasis, renal cell carcinoma	Paraffin section	810	110	76	402–612	N/A	
Pancreas	Xu et al. ^92^	Ductal adenocarcinoma	Frozen section	810	110	76	430–716	389–410	
	Chen et al. ^106^	Pancreatic neoplasm	Paraffin section	810	110	76	350–710	N/A	
Uterus	Wang et al. ^34^	Ovarian cancer	Frozen section	810	140	80	N/A	395–415	
	Qian et al. ^107^	Epithelial ovarian cancer	Frozen section	810	140	80	N/A	390–420	
	Pouli et al. ^108^	Squamous intraepithelial lesions	Resected tissue	755, 860	150	80	440–480, 500–550	N/A	
	Pouli et al. ^94^	Ovarian malignancy	Resected tissue	755, 900	N/A	N/A	500–550	440–480	
	Wen et al. ^31^	High-grade serous ovarian cancer	Frozen section	890	100	N/A	N/A	435–455	
Bladder	Pradère et al. ^109^	Urothelial carcinoma	Resected tissue	870	N/A	N/A	502.5–537.5	438–458	
	Jain et al. ^88^	Urothelial carcinoma in situ	Resected tissue	780	160	N/A	420–490	360–400	
	Jain et al. ^110^	Bladder tumor	Resected tissue	780	160	N/A	420–530	355–420	
Prostate	Ling et al. ^93^	Prostate cancer	Paraffin section	880	N/A	N/A	N/A	430–450	
	Huland et al. ^111^	Prostate cancer	Resected tissue	800	150	50	405–700	<405	
Skin	Huttunen et al. ^112^	Squamous cell carcinoma	Paraffin section	860	140	80	440–490, 510–600	425–435	
	Arginelli et al. ^113^	Melanocytic nevi	Resected tissue	760, 800	75	80	N/A	N/A	
	Seidenari et al. ^114^	Basal cell carcinoma	Resected tissue	760, 820	75	80	N/A	N/A	
**(b)**									
Brain	Wang et al. ^115^	Epilepsy	Frozen section	810	140	80	428–695	389–419	1. **Cell morphology:** atypia, dyskeratosis, apoptosis, different shapes and sizes, larger nuclei, basal cells, epithelial cells, goblet cells, foveolar gastric mucous neck cells, parietal cells, chief cells, flask-shaped cells, lipofuscin, red blood cell congruence. 2. **Tissue structure:** abnormal radial cortical lamination, piamater, occasional polymorphonuclear inflammatory cells infiltrating into the squamous epithelium, the height of connective tissue papillae was increased, the general tissue structure of the airway and elastin structures, steatosis, epidermal pleomorphism and spongiosis, epidermal thickness and pigmentation increased, intercellular oedema and an impaired architecture. 3. **Stroma:** the network-like structure of elastin, unique features of linear structure and dense elastin, vascular septa rich in elastic fibers. Curly collagen fibers, disorganized and not exclusively intermingled with the collagen fibers, the diffuse and focal accumulation of collagen, tight collagen networks, disrupted and fragmented collagen fibers.
Eye	Batista et al. ^116^	Keratoconus	Resected tissue	800	10	85	425–575	400–425	
Artery	Pukaluk et al. ^96^	Atherosclerotic lesion	Resected tissue	880	N/A	N/A	500–550	435–485	
	JAIN et al. ^117^	Atherosclerotic plaques	Resected tissue, paraffin section	780	N/A	N/A	420–490 550–650	360–400	
Esophagus	Wong et al. ^118^	Barrett esophagus	Resected tissue	735, 800	N/A	N/A	420–680	390–410	
Lung	Kottmann et al. ^119^	Interstitial pneumonia	Paraffin section	810	100	80	N/A	390–420	
	Tilbury et al. ^120^	Idiopathic pulmonary fibrosis	Resected tissue	890	N/A	N/A	572–594	435–455	
	Tjin et al. ^121^	Chronic obstructive pulmonary disease	Paraffin section	810	100–200	N/A	565–605	400–410	
Heart	Yang et al. ^97^	Cardiomyopathy	Paraffin section	810	140	80	428 –677	395–415	
	Zhang et al. ^122^	Coronary atherosclerotic plaque	Resected tissue Paraffin section	790	N/A	N/A	425–495	382.5–407.5	
Liver	Goh et al. ^123^	Chronic hepatitis	Paraffin section	780	N/A	N/A	506–594	384.5–395.5	
	Yan et al. ^79^	Liver cirrhosis	Resected tissue	800	110	76	430–708	387–409	
Skin	Wang et al. ^28^	Morphea, lichen sclerosus	Paraffin section	830	140	80	438–695	405–425	
	Meng et al. ^39^	Keloid scar	Frozen section	700–980	110	76	430–697	398–409	
	Jiang et al. ^124^	Hypertrophic scar	Frozen section	850	110	76	457–714	414–436	
	Han et al. ^125^	Pathological scar	In vivo	780	1560	80	420–580	375–400	
	Utino et al. ^126^	Tuberculoid leprosy, sarcoidosis	Paraffin section	800	<100	N/A	N/A	370–410	
	Springer et al. ^127^	Acute wound	In vivo	790	N/A	N/A	N/A	N/A	
	Huck et al. ^128^	Atopic dermatitis	In vivo	710	100	N/A	430–490	N/A	
	Koehler et al. ^129^	Typical epidermal changes induced by acute UVB irradiation	In vivo	790	N/A	N/A	410–490	N/A	
	Tong et al. ^130^	Solar elastosis, anetoderma, striae distensae	Frozen section, paraffin section	900	N/A	N/A	490–520	437–497	
	Lin et al. ^131^	Discoid lupus erythematosus	Frozen section	810	110	76	430–697	398–409	

## AI-empowered multiphoton pathological diagnosis

**Fig. 4:**
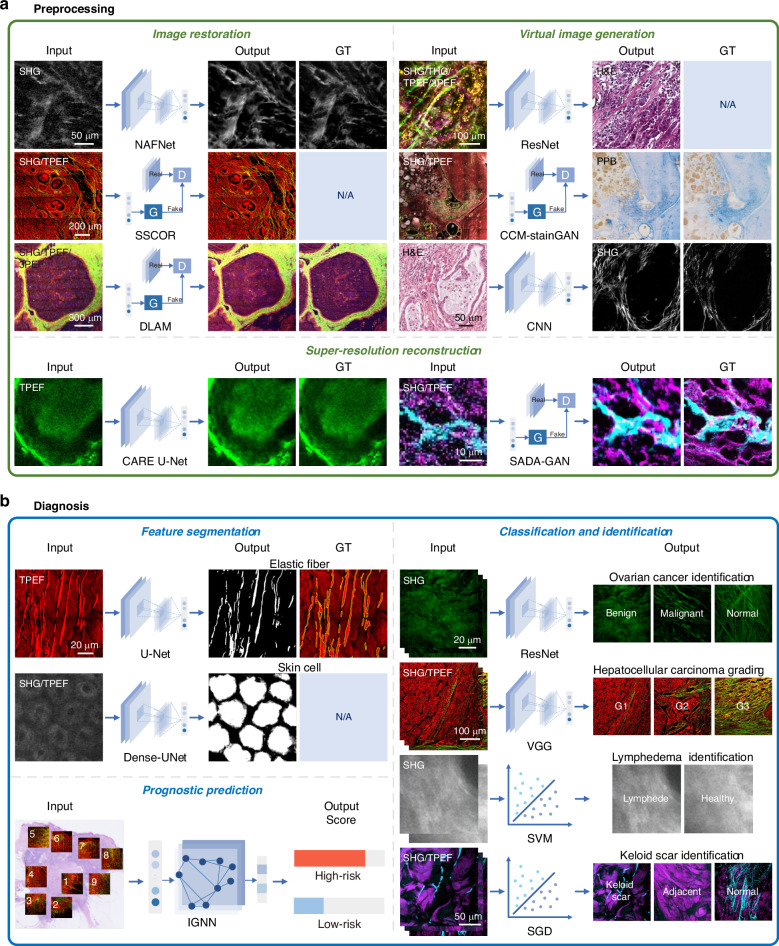
Representative multiphoton pathological diagnosis algorithms. **a** Preprocessing is a necessary step to improve the accuracy of downstream diagnostic tasks, which is categorized into three types: virtual staining^17,140,141^, image restoration^73,134,135^, and super-resolution reconstruction^136,138^. **b** Diagnosis is divided into three categories: feature segmentation^28,29^, classification and identification^33–35,39^, and prognosis prediction^42^. Classification and identification are further subdivided into using deep learning models and traditional machine learning models. These categories encompass the input mode, model type, and output result. Adapted with permission from ref. ^33,138,140^ © Optical Society of America

Prior to the application of AI in multiphoton images, conventional digital image processing algorithms, such as collagen fiber analysis^132^ and saliency detection^133^, were already in existence for the quantitative assessment of tumor-specific multiphoton diagnostic features. In this section, as shown in Fig. 4, we specifically focus on the work related to machine learning and deep learning, providing a brief overview of their empowered capabilities in pathological diagnosis, including image preprocessing, disease diagnosis, and prognosis prediction.

The quality of multiphoton images serves as a prerequisite for ensuring the accuracy of disease diagnosis and prognosis prediction. Therefore, before using multiphoton images for disease diagnosis, researchers often employ image preprocessing techniques, such as image restoration^73,134,135^ and image super-resolution models^136–138^, to enhance the image textural details and restore hidden pathological features. For instance, to address image quality caused by uneven sample or system instability, adaptive sampling driven by the uncertainty of predicted pixels can be employed to reduce noise^134^. For stitched multiphoton images, stripe self-correction networks based on proximity sampling scheme can effectively correct stripes or artifacts in the stitched positions^135^. Additionally, a self-alignment dual-attention-guided super-resolution network can produce high-quality multiphoton images while mitigating the risk of photobleaching^138^. These preprocessed high-resolution, high-contrast multiphoton images can further enhance the accuracy of downstream diagnostic tasks, such as cell segmentation and counting, and improve the precision of prognostic tasks related to the extraction of collagen features.

On the other hand, as multiphoton imaging gradually enters the field of pathology, virtual image generation techniques serve as a complementary form of preprocessing that enhances the acceptance of multiphoton pathology images. Virtual image generation encompasses the transformation from label-free multiphoton images to virtual pathological staining images^17,72,139,140^, as well as the generation of virtual multiphoton images from H&E-stained images^141^. For instance, virtual staining models based on generative adversarial networks (GANs)^17^ or convolutional neural networks (CNNs)^72,139–141^ can transform multi-channel multiphoton images into H&E staining or specific staining images. Although virtual staining images may sometimes deviate in detail from real stained images, these pathology-styled images assist pathologists in interpreting multiphoton images more effectively. Moreover, CNN architectures with pixel-shuffle layers can generate virtual SHG images directly from H&E-stained images^141^, eliminating the need for additional staining agents or equipment. This provides a cost-effective method for quantitatively extracting collagen fiber directionality and alignment features. These preprocessing steps provide the foundation for subsequent pathological analysis, facilitating more accurate diagnosis and prognosis.

Currently, diagnostic challenges persist in contemporary pathology, with specific scenarios proving particularly difficult to interpret with precision. Notable examples include the differentiation between glioblastoma and primary lymphoma^142^, and between ductal carcinoma in situ and microinvasive carcinoma of the breast^82^. These diagnostic challenges share similarities, often cannot be addressed through conventional pathological techniques. For instance, H&E staining struggles to differentiate or accurately quantify vascular-related elastic fibers and collagen fibers. Additionally, specialized cells such as myoepithelial and basal cells are prone to confusion with neighboring proliferative fibroblasts in the stroma. Excitingly, MPM offers a promising solution that aids in the identification of ambiguous cells, which helps mitigate the subjective differences among pathologists. More importantly, the integration of AI introduces a level of objectivity, supplying auxiliary information that enhances the subjective visual diagnosis performed by human experts. Researchers commonly utilize machine learning methods and deep learning models to automatically extract distinct features of cellular cytoplasm and extracellular matrix from multiphoton images. For instance, segmentation models based on U-Net are employed to extract multiphoton features such as elastic fibers^28^ and cells^29^, enabling rapid detection and quantification of pathological regions. Besides, a novel diagnostic method has been developed by fusing the H&E segmentation results of cell nuclei with multiphoton images, leading to more accurate diagnoses of microinvasion in ductal carcinoma in situ^82^. The method of combining feature extraction methods with machine learning classifiers, such as support vector machine (SVM)^33^ or stochastic gradient descent (SGD)^30^ classifier, has shown superior performance in classifying diseases, particularly on small datasets. In contrast, using deep learning classification networks such as ResNet^34^ or VGG^35^ allows for the automatic learning of complex pattern.

Prognostic prediction is of paramount significance for understanding disease progression and guiding patient treatment. A robust prognostic prediction model is often associated with the accuracy of pathological diagnostic results and the discovery of pathological novel insights. For instance, based on the tumor-associated collagen signature patterns revealed by MPM in invasive breast cancer, the integration of graph neural networks has facilitated a deeper interpretation of the spatial distribution of these patterns in tumor development^42^. This approach also provides new clues for the precise classification and treatment of different breast cancer subtypes. With the gradual accumulation of multiphoton datasets, AI-empowered MPM augments the dimensions and efficiency of traditional pathology, elevating multiphoton-assisted diagnosis to a more intelligent and precise level, thereby assisting clinicians in improving the diagnostic accuracy of intractable cases. Table 3 provides a detailed summary of the model types, inputs, and outputs involved in representative label-free multiphoton image preprocessing and intelligent pathological diagnosis from 2013 to 2023^17,28–42,72,73,134–141^.

**Table 3:** (a) Image preprocessing algorithms for multiphoton microscopy. (b) Diagnosis algorithms for multiphoton microscopy

(a)							
Task	Authors	Input		Output	Model	Supervised/ Unsupervised	Model evaluation
		Modalities	Image size(pixel)				
Image restoration	Ye et al. ^134^	SHG	512 × 512	Low noise images with partial regions rescanned	NAFNet	Supervised	SSIM, MSE
	Shen et al. ^73^	SHG/TPEF/ 3PEF	256×256 2176 × 2176	The persistent noise, distortions, and scanning fringes reduced images	DLAM	Supervised	PSNR, SSIM
	Wang et al. ^135^	SHG/TPEF	>3000 × 3000	Stripe and artifact corrected images	SSCOR	Unsupervised	PSNR, SSIM, ICV
Virtual image generation	Picon et al. ^139^	TPEF	448 × 448	H&E	Dense-UNet	Supervised	Diagnostic F1
	Keikhosravi et al. ^141^	H&E	128 × 128	SHG	CNN	Supervised	PSNR, SSIM
	Wang et al. ^17^	SHG/TPEF	256 × 256 512 × 512 1024 × 1024 2048 × 2048	H&E/PPB	CCM-stainGAN	Unsupervised	Accuracy
	Borhani et al. ^72^	TPEF	300 × 300 16 × 16	H&E	FCNN-p2p VGG-a2p	Supervised	NMSE, SSIM
	Shi et al. ^140^	SHG/THG/ TPEF/3PEF	256 × 256	H&E	MM-MIL	Supervised	Diagnostic AUC
Super-resolution reconstruction	McAleer et al. ^136^	TPEF	128 × 128	High resolution images	U-Net	Supervised	SSIM, MSE
	Lin et al. ^137^	SHG/TPEF	24 × 24	High resolution images	ResNet	Supervised	PSNR, SSIM
	Zhao et al. ^138^	SHG/TPEF	10240 × 10240 2176 × 2176 1024 × 1024	High resolution images	SADA-GAN	Unsupervised	PSNR, SSIM

(b)									
Task	Authors	Input		Output	Disease	Model	Supervised/ Unsupervised	Model evaluation	Accuracy
		Modalities	Image size(pixel)						
Feature segmentation	Wang et al. ^28^	TPEF	256 × 256 × 4	Elastic fiber segmentation	Lichen sclerosus, morphea	U-Net	Supervised	Accuracy, mIoU, mPA, F1	85.2%
	Cai et al. ^29^	SHG/TPEF	128 × 128	Skin cells segmentation	Skin disease	Dense-UNet	Supervised	Accuracy, Dice, F1	92.5%
Classification and identification	Terradillos et al. ^30^	TPEF	299 × 299 × 3	Malignant neoplastic colon lesions, healthy, hyperplastic, benign neoplastic tissue	Colorectal cancer	Xception	Supervised	Accuracy, Sensitivity, Specificity, SD, PPV, NPV, SCP	86.7%
	Wen et al. ^31^	SHG	49 × 49	Normal tissue, high grade malignant tissue	Ovarian cancer	Kmeans	Supervised	Accuracy, AUROC	97.0%
	Wen et al. ^32^	SHG	50 × 50 × 5	Normal, high risk ovarian stroma, benign ovarian tumors, low grade, high grade serous tumors, endometrioid tumors	Ovarian cancer	Kmeans	Supervised	Accuracy, AUROC	87.1%
	Kistenev et al. ^33^	SHG	32 × 32	Lymphedema tissue, healthy tissue	Lymphedema	SVM	Supervised	Accuracy, Sensitivity, Specificity	96.0%
	Wang et al. ^34^	SHG	224 × 224	Normal, benign, malignant ovarian tissue	Ovarian cancer	ResNet	Supervised	Accuracy	99.7%
	Lin et al. ^35^	SHG/TPEF	224 × 224	G1(well-differentiated), G2(moderately differentiated), G3(poorly differentiated), G4(undifferentiated)	Hepatocellular carcinoma	VGG	Supervised	Accuracy, AUC	94.00%
	Yang et al. ^36^	SHG/TPEF	256 × 256	Epithelium segmentation images of prostate tissues: benign, Gleason pattern 3, Gleason pattern 4, Gleason pattern 5	Prostate cancer	U-Net AlexNet	Supervised	Classification accuracy, Segmentation pixel accuracy	81.1% (classification) 92.3% (segmentation)
	Xi et al. ^38^	SHG/TPEF	256 × 256	G1(well differentiated), G2 (moderately differentiated), G3 (poorly differentiated)	Breast cancer	GAN	Unsupervised	Accuracy, AUC	90.0%
	Huang et al. ^37^	SHG/TPEF	224 × 224	Images labeled tumor regions	Liver cancer	ResNet	Supervised	Accuracy	99.8%
	Meng et al. ^39^	SHG/TPEF	N/A	Normal, scar, adjacent tissues	Keloid scar	SGD Classifier	Supervised	Accuracy, AUC	96.2%
	Blokker et al. ^40^	THG	1000 × 1000	Glioma, non-tumor tissue	Gliomas	FCN	Supervised	Accuracy, AUC	79.0%
	You et al. ^41^	SHG/THG/ TPEF/3PEF	256 × 256 × 4	Cancer, normal tissue	Breast cancer	ResNet InceptionV3	Supervised	Accuracy, AUC	97.0%
Prognostic prediction	Qiu et al. ^42^	SHG/TPEF	N/A	Prognosis score	Breast cancer	IGNN	Unsupervised	AUC	N/A

## Challenges and future perspectives

**Fig. 5:**
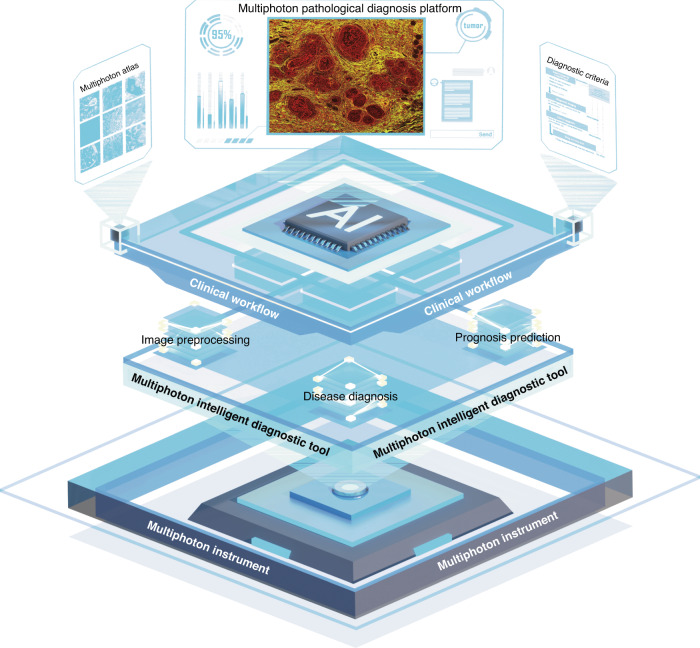
Structural diagram of AI-empowered multiphoton pathology. The multiphoton instrument and multiphoton dataset serve as the cornerstone of multiphoton pathological diagnosis, laying the foundation for the development of intelligent diagnostic tools. These tools are developed based on advanced model architectures and training paradigms, possessing functions such as image preprocessing, disease diagnosis, and prognosis prediction, ultimately integrated into an intelligent diagnostic platform. By integrating the multiphoton atlas and diagnostic criteria into clinical workflow, AI-empowered multiphoton pathology promotes the establishment of a paradigm for multiphoton pathological diagnosis

AI-enhanced multiphoton pathology has markedly advanced the integration of multiphoton microscopes into practical clinical settings, and intelligent multiphoton pathology diagnosis has reached a comparable performance to human experts in certain tasks such as conventional H&E diagnostics. However, only a handful of these algorithms have been successfully integrated into standard clinical processes. As a result, the realization of effective artificial intelligence-based pathology diagnostics using multiphoton imagery remains fraught with challenges. As illustrated in Fig. 5, we will address these obstacles specifically associated with intelligent multiphoton pathology, examining the aspects of multiphoton imaging technologies, dataset acquisition, the development of deep learning algorithms, and their integration into clinical diagnostic procedures.

### Multiphoton digital pathological diagnostic instrument

#### High-speed and high-throughput capability

In comparison to clinical digital slide scanners, current multiphoton microscopes are still limited by imaging speed, cost, and image field of view. Particularly, there is an urgent need for a novel pathology imaging instrument with high-speed and high-throughput capabilities, similar to that of a digital slide scanner. The imaging speed of current multiphoton microscopes is primarily attributed to the scanning speed of two-axis mechanical scanning mirrors and the precision of motorized positioning stages. However, compared to digital slide scanner, the primary challenge lies in the sample preparation process for unstained slices used in multiphoton imaging. This process needs further optimization, such as adjusting slice thickness and adhering to sealing standards. Therefore, it is essential to standardize the quality of label-free slices based on objective working distance or excitation power of laser. This standardization is a prerequisite for improving the imaging throughput of multiphoton instruments. On the other hand, high-throughput simultaneously presents challenges in data transfer and storage. Currently, there are variations in data formats of commercial microscopes from different companies. Consequently, standardizing the multiphoton file formats for specific image compression protocols not only facilitates large-scale data storage but also promotes image sharing and consultation among pathologists.

#### Miniaturized and portable design

Research-grade multiphoton imaging instruments typically feature multifunctional characteristics, such as tunable femtosecond lasers, high-resolution spectrometers, and directly viewable eyepieces. However, some of features may be redundant for clinical applications. Therefore, multiphoton microscopes tailored for clinical pathology need to simplify the functionalities of research-grade desktop multiphoton microscopes. This simplification not only enhances system usability but also effectively reduces the overall system size, weight, and manufacturing costs. It also makes multiphoton pathology microscopes more affordable and accessible to a broader range of medical centers and researchers, promoting their global adoption. Furthermore, miniaturized multiphoton pathology microscopes offer enhanced mobility and flexibility. In contrast to large-scale research-grade equipment, portable multiphoton microscopes do not require specific environments like cleanrooms for operation. This makes them more suitable for various clinical environments and applications, such as postoperative diagnosis in pathology departments, rapid intraoperative diagnosis in operating rooms, and bedside diagnosis in hospital wards. Notably, compared to conventional optical microscopes in pathology departments, the expense of miniaturized multiphoton microscopes remains considerable. This is primarily attributed to the costs associated with precision equipment, including femtosecond lasers, high numerical aperture objectives, and photomultiplier tubes. Therefore, although miniaturization facilitates the clinical integration of MPM, the substantial upfront investment and ongoing maintenance expenses frequently influence hospitals’ procurement decisions. Such factors may impede the widespread adoption and collaborative utilization of MPM within medical institutions, ultimately diminishing its overall equipment utilization rates.

#### Multi-modality functionality

In clinical decision-making, the comprehensive utilization of multi-modal information is crucial for a more holistic understanding of diseases, encompassing clinical data and the combination of different imaging modalities such as radiology and pathology. For multiphoton microscopes, in addition to the four nonlinear optical effects (SHG, THG, 2PEF, 3PEF), SRS and coherent anti-stokes Raman scattering (CARS) also exhibits high specificity for different types of biomolecules. The integration of MPM and SRS/CARS in multimodal microscopy enables a more comprehensive characterization of the distribution of pathological features in tissues^143^. This not only aids in the discovery of novel pathological markers but also provides insights into disease mechanisms. On the other hand, H&E staining are one of the most used diagnostic tools in pathology. Encouragingly, H&E-stained specimen can also be excited to produce multiphoton signals. Therefore, if H&E-stained imaging can be integrated with multiphoton imaging at the image or instrument level, it not only provides more comprehensive information during diagnosis but also enhances the reliability and accuracy of pathological diagnosis. Importantly, the integration of H&E staining establishes a more solid foundation for the widespread application of multiphoton pathology instruments in clinical settings.

### Task-oriented high-quality open-source multiphoton datasets

#### Focusing on specific clinical tasks

As multiphoton instruments are not yet widely employed in clinical pathology, the current scale of multiphoton image datasets is far smaller than that of digital pathology datasets. However, the effectiveness and utility of datasets are prerequisites for expanding dataset scale. To harness the unique advantages of multiphoton pathology diagnosis, it is imperative to establish task-oriented multiphoton pathology datasets, such as those for distinguishing brain tumors from pituitary tumors. Driven by specific clinical tasks, surgeons, pathologists, microscopists, and computer engineers need to collaboratively plan inclusion criteria, case numbers, image dimensions, annotation rules from the early stages of model development. This collaboration is essential to avoid biases that may impact model training. These task-oriented multiphoton datasets not only attract computer vision researchers to improve model metrics, but also draw more attention from clinical practitioners to the auxiliary diagnostic potential of MPM.

#### Image quality of dataset

Due to influences from factors such as photomultiplier gain, laser power, and sample preparation quality, even the same tissue slices may exhibit resolution and color discrepancies in images scanned by different multiphoton instruments. Such differences in image quality pose challenges to the transferability of the same model between two seemingly similar multiphoton datasets. Despite the development of some style normalization or style transfer models, these models often achieve optimal performance only on specific datasets. Therefore, AI-assisted multiphoton pathology diagnosis should emphasize the rationalization of imaging parameters, standardization of imaging processes and specimen preparation. By exploring and establishing a consensus on the entire process from specimen to imaging, we may be able to control the quality of multiphoton image data from the source, thus addressing the generalization gap caused by inherent heterogeneity in histopathological data.

#### Open sourcing and sharing of dataset

Currently, acquiring multiphoton image still faces challenges, primarily due to the high academic value of multiphoton datasets and legal or ethical constraints involving human samples. It is worth noting that the rapid development of computer vision is closely related to the open-source and large-scale natural image datasets. To further propel the impact of multiphoton-assisted diagnosis, high-quality work should proactively release the datasets required for training models as much as possible, especially training data. This will prevent researchers from overestimating the performance of the models. Furthermore, to promote the sharing of large-scale datasets, we need to establish a network platform supporting online preview and download of multiphoton image data. This platform should include raw data, corresponding pathological images, dataset descriptions, and task instructions. On the other hand, to address challenges in sharing data when constructing multicenter datasets across different countries due to ethical and regulatory obstacles, federated learning and swarm learning can be attempted to jointly train the models. Federated learning allows multiple institutions to collaboratively improve a global model while preserving the confidentiality of their individual data sets. In parallel, swarm learning enhances prediction accuracy and robustness by integrating diverse models. This approach effectively mitigates overfitting and enhances the model’s generalization capabilities.

### Custom-developed multiphoton deep-learned diagnostic tool

#### Transitioning from supervised to unsupervised training paradigm

Supervised, unsupervised, and semi-supervised learning are the three main training paradigms in deep learning. Supervised learning relies on experts annotating multiphoton images, but obtaining paired ground truth can be challenging. Computational constraints often lead to training gigapixel or terapixel-level images with annotated patches, which is time-consuming and expensive. Moreover, models trained on a single dataset usually lack strong generalization. Self-supervised learning addresses this by designing supervision tasks that transform unsupervised learning into a supervised problem without requiring manual annotations, while semi-supervised learning leverages a small amount of labeled data alongside unlabeled data to reduce dependency on extensive labeling. In segmentation tasks, a self-supervised domain adaptation framework, based on target-specific fine-tuning, adapts the original model to different target-specific pathological tissues for cell segmentation. This domain adaptation occurs across various tissues and multiple medical centers without accessing the source dataset, enhancing the model’s performance even with minimal labeled data^144^. Additionally, a semi-supervised semantic segmentation network, SCANet, based on a three-branch architecture, alternately trains a multi-scale recurrent neural network branch, a consistency decoder branch, and an adversarial learning branch. This achieves excellent segmentation performance with a small amount of labeled data and extensive unlabeled data^145^.

Weakly supervised learning harnesses imprecise or incomplete weak label information to train models, mapping input data to stronger labels, thereby reducing reliance on precise annotations. In classification tasks, a weakly supervised learning framework using Information Bottleneck theory fine-tunes the backbone to create task-specific representations from WSI-level weak labels, addressing the limited annotation issue in pathological image classification^146^. Similarly, another weakly supervised learning framework based on RankMix data augmentation, adapts sample quantities in the training set according to task contributions and mix images of different sizes, mitigating issues of data scarcity and class imbalance^147^. Ultimately, self-supervised or weakly supervised learning holds promise in addressing challenges such as inadequate generalization, data scarcity, and insufficient labeled data in multiphoton pathology models.

#### Model architecture for general intelligence

Model performance metrics reflect their ability to perform tasks on specific datasets. From a diagnostic perspective, pathologists are equally concerned about the intelligence of the model’s adaptability and handling of boundary conditions. Firstly, introducing advanced model architecture is crucial for the future of multiphoton intelligent diagnostics. Unlike CNN models trained on small patient cohorts, combining a pre-trained encoder with a transformer network for patch aggregation has been validated for end-to-end biomarker prediction on a large multicenter cohort of over 13,000 colorectal cancer patients^148^. On the other hand, the process of multiphoton imaging is interpretable, where the pixel intensity in the image represents the spectral characteristics of endogenous fluorescence signal sources. Thus, incorporating the physical principles of MPM into the model ensures more effective capture of endogenous information, potentially revolutionizing the interpretation of multiphoton data and enhancing both generalizability and efficiency.

Secondly, a single modality often fails to fully reveal the complex mechanisms and diversity of diseases, medical centers have established multidisciplinary teams for the clinical treatment of major illnesses. Moreover, molecular pathology laboratories equipped with technologies such as genetic testing, protein analysis, and fluorescence imaging are increasingly demonstrating their capacity for precise diagnosis. Therefore, AI models that integrate multimodal data can provide comprehensive and scientifically sound diagnostic decisions. The histological and genomic features are extracted using a multiple instance learning network and a self-normalizing network, followed by feature fusion through Kronecker product integration to achieve cancer prognosis prediction^149^. The iStar model, which is based on hierarchical image feature extraction, combines spatial transcriptomics data with high-resolution histological images to predict super-resolution spatial gene expression^150^. Pan-cancer computational histopathology represents image tile as 1536-dimensional vectors and uses high-dimensional regression methods to integrate histological, genomic, and transcriptomic features, accurately discriminates 28 cancer and 14 normal tissue types^151^. As a result, incorporating multiphoton image features into the multimodal AI models has the potential to offer unique new perspectives on the interactions between cells and the extracellular matrix within the tumor microenvironment.

Finally, foundational models like ChatGPT in natural language processing demonstrate capabilities for general intelligence, facilitating the development of multiphoton diagnostic models with multitasking abilities. Future advancements will enable tasks such as transforming between multiphoton and H&E images, interpreting multiphoton images alongside pathological reports, and engaging in iterative question-and-answer sessions involving pathological findings and doctor-patient interactions^152^. A more challenging prospect is transforming multiphoton microscopes into intelligent entities through specialized models, allowing interaction with high-throughput images in clinical pathology diagnostics. This embodied intelligent learning paradigm will ultimately lead to new emergences in MPM diagnostic capabilities, providing opportunities to construct a general intelligent model adaptive to diseases.

#### Interpretability, repeatability and reliability

Although some multiphoton diagnostic models perform exceptionally well on datasets, even matching or surpassing human diagnostic tasks, the primary hurdle in clinical application is the “black-box” nature of deep learning, i.e., lack of interpretability. Pathologists express concern about writing diagnostic reports when they lack an understanding of how the model reaches its conclusions. Despite interpretability of neural networks has been a long-standing challenge, the methods like feature visualization could provide an approximate explanation of the model’s working process. These visualization results enhance pathologists’ trust in model-assisted decision-making. On the other hand, the reliability of auxiliary diagnostics is also reflected in the model’s repeatability. In the field of multiphoton medicine, while extensive work has been done on medical statistical analysis or model ablation experiments, open-source code contributions are limited. For the open-source work, the training weights of the model are particularly crucial for reproducing results. Therefore, if we verify the repeatability of the model through sufficient code access privileges and data resources, thereby providing confidence intervals, capability boundaries, and computational consumption. This will increase the reliability of the model for clinical deployment. However, achieving breakthroughs in AI interpretability poses significant challenges in the short term. If guided by outcome-driven assessments of model feasibility, clinical validation of deep learning methods emerges as a crucial pathway to enhancing AI reliability, particularly in healthcare settings. For instance, within large-scale multicenter trials employing AI-empowered MPM, despite lingering uncertainties regarding the interpretability of the models, the accuracy metrics of diagnostic tasks serve as robust indicators of their reliability and stability. This, in turn, will also enhance patient acceptance of this novel technology.

### The clinical workflow of integrated multiphoton pathology

#### Multiphoton pathological diagnostic criteria

Pathologists, drawing upon years of accumulated knowledge and experience, have established standard criteria for conventional pathological diagnosis. Even though MPM has demonstrated a series of advancements in pathological applications, firstly, it is essential to establish atlases tailored to multiphoton diagnosis. These atlases should elucidate the diverse applications of multiphoton images across various pathological scenarios. They ought to encompass multiphoton images alongside corresponding images of fresh tissue, frozen sections, paraffin-embedded sections, smears, and organoids for comparative reference. Such comprehensive coverage will assist pathologists in gaining deeper insights into MPM indications and serve as an introductory guide for computer vision researchers exploring multiphoton imaging. Secondly, pathologists typically have minimal or no training in the use of multiphoton-assisted diagnostic technologies. To facilitate a rapid understanding of multiphoton images by pathologists, a virtual staining model can be employed. Multiphoton images can be transformed into virtual H&E images, special stains, and even holds the potential for conversion into immunohistochemistry or immunofluorescence images^153^. This capability allows pathologists to engage in paired comparative learning, assisting them in gradually incorporating multiphoton features into their diagnostic workflow. With the growing trust among pathologists in multiphoton diagnostics, multi-center clinicians can continuously validate and explore new multiphoton features in clinical practice. This iterative process allows for the enhancement of multiphoton diagnostic capabilities across various medical settings. Finally, combined with efficient AI analysis, this approach can further aid in formulating comprehensive pathology workflows and improving diagnostic precision. For instance, Pohlkamp et al. investigated the use of machine learning to support microscopic differential counts of peripheral blood smears within a high-throughput hematology laboratory setting^154^. Nasrallah et al. utilized machine learning for cryosection pathology to predict the 2021 WHO classification of glioma^155^. As multiphoton diagnostic methods achieve consensus, it is anticipated that clinicians and imaging experts will collaboratively integrate multiphoton diagnostic features into clinical diagnostic guidelines or novel histological grading systems for specific diseases.

#### Multiphoton pathological diagnosis platform

The prospect of multiphoton AI-assisted diagnostic algorithms is exciting for pathologists. However, pathologists typically a background in computer science, and reproducing algorithms or configuring environments can be labor-intensive for them. In the pathology diagnostic workflow, pathologists prefer “plug-and-play” intelligent diagnostic software for decision support. Therefore, there is an urgent need to integrate mature multiphoton diagnostic algorithms into pathology diagnostic systems, such as picture archiving and communication system, in the form of software packages. Pathologists can seamlessly import MPM-based diagnostic results into conventional diagnostic reports, facilitating easier integration into existing diagnostic workflows. Additionally, using cloud-based interactions, pathologists can collaboratively assess this novel multiphoton pathology report with colleagues, with final decisions made by senior pathologists. For less mature models, there is a need for a research-level specialized diagnostic platform for multiphoton images, similar to DeepImageJ^156^. This platform should deploy and fine-tune pre-trained deep learning models, creating a library of multiphoton diagnostic algorithms. Based on various postoperative or intraoperative diagnostic requirements, algorithms from the platform can be selectively deployed to edge or cloud servers. Besides, a feedback mechanism should be incorporated into the software process to iteratively optimize the diagnostic performance of algorithms in clinical trials.

#### Ethical security and AI risks

While interdisciplinary personnel have considered ethical security concerns in constructing multiphoton image datasets, multiphoton images involve patient privacy information. In the data management and analysis processes of multiphoton diagnostic software, apart from pathologists, it may also involve bioinformatics, statistics, and computer vision researchers. This may inadvertently lead to risks related to personal privacy or the illegal utilization of data. To address these issues, it is essential to establish privacy protection mechanisms and data protection regulations concerning multiphoton-related data. Beyond ethical security, AI risks also demand attention. Data poisoning and adversarial sample attacks are common methods that threaten the security of the model. Data poisoning involves injecting malicious samples or altering data in the training set to deceive the model, leading to incorrect predictions in future tasks. Adversarial attacks, on the other hand, subtly but purposefully modify input data to cause the model to produce incorrect results. The augmentation of multiphoton data significantly enhances the model’s inference capabilities. However, this increased capability may introduce false features that clinicians find challenging to identify. For instance, the results of virtual staining, without a ground truth for comparison, already poses challenges for pathologists in distinguishing between authentic and synthetic information. This uncertainty can introduce decision biases for pathologists, with the impact on patient prognosis difficult to estimate. To mitigate these AI risks and reduce uncertainty in diagnosis, perhaps there is no need to blindly pursue the innovation and performance of the model; instead, emphasis should be placed on the practicality and stability of the model. Additionally, multiphoton diagnostic software requires rigorous clinical validation and regulatory approval. Through randomized clinical trials, it can determine the role of multiphoton diagnostic algorithms in the entire diagnostic workflow. This ensures the provision of more reliable, controllable, and secure diagnostic results.

## Conclusion

Despite the hurdles in progressing multiphoton microscopy (MPM) from traditional pathological uses to intelligent diagnostics, the movement toward smart multiphoton pathology is actively underway. As multiphoton pathology tools evolve and the collection of relevant datasets grows, we anticipate a marked enhancement in both the breadth and depth of artificial intelligence applications within this field. Pathologists are beginning to grasp the enhanced capabilities offered by multiphoton technology. However, it is important to stress that the successful implementation of such sophisticated technology hinges on synchronized collaborative efforts from diverse, interdisciplinary teams across multiple centers. This cooperation is vital for turning scientific discoveries into actionable diagnostic criteria, for refining early-stage prototypes into approved medical devices, and for evolving open-source algorithms into accessible, user-centered software interfaces. With these concerted efforts, MPM is poised to become a cornerstone in the future landscape of diagnostic pathology.
